# Effect of Amino-Functionalized Polyhedral Oligomeric Silsesquioxanes on Structure-Property Relationships of Thermostable Hybrid Cyanate Ester Resin Based Nanocomposites

**DOI:** 10.3390/polym15244654

**Published:** 2023-12-09

**Authors:** Olga Grigoryeva, Alexander Fainleib, Olga Starostenko, Diana Shulzhenko, Agustin Rios de Anda, Fabrice Gouanve, Eliane Espuche, Daniel Grande

**Affiliations:** 1 Institute of Macromolecular Chemistry, National Academy of Sciences of Ukraine, 48, Kharkivske Shose, 02155 Kyiv, Ukraine; fainleib@i.ua (A.F.); o_starostenko@ukr.net (O.S.); darkblue1cherry2black@gmail.com (D.S.); 2 Université Paris Est Creteil, CNRS, Institut de Chimie et des Matériaux Paris-Est, UMR 7182, 2 rue Henri Dunant, 94320 Thiais, France; agustin.rios-de-anda@u-pec.fr (A.R.d.A.); daniel.grande@cnrs.fr (D.G.); 3 Université Claude Bernard Lyon 1, CNRS, Ingénierie des Matériaux Polymères, UMR 5223, 15 Boulevard André Latarjet, 69622 Villeurbanne, France; fabrice.gouanve@univ-lyon1.fr (F.G.);

**Keywords:** cyanate ester resin, amino-POSS, polycyanurate, glass transition temperature, thermal stability, gas permeability

## Abstract

Nanocomposites of cyanate ester resin (CER) filled with three different reactive amino-functionalized polyhedral oligomeric silsesquioxane (POSS) were synthesized and characterized. The addition of a small quantity (0.1 wt.%) of amino-POSS chemically grafted to the CER network led to the increasing thermal stability of the CER matrix by 12–15 °C, depending on the type of amino-POSS. A significant increase of the glass transition temperature, *T*_g_ (DSC data), and the temperature of α relaxation, *T*_α_ (DMTA data), by 45–55 °C of the CER matrix with loading of nanofillers was evidenced. CER/POSS films exhibited a higher storage modulus than that of neat CER in the temperature range investigated. It was evidenced that CER/aminopropylisobutyl (APIB)-POSS, CER/*N*-phenylaminopropyl (*N*PAP)-POSS, and CER/aminoethyl aminopropylisobutyl (AEAPIB)-POSS nanocomposites induced a more homogenous α relaxation phenomenon with higher *T_α_* values and an enhanced nanocomposite elastic behavior. The value of the storage modulus, *E*′, at 25 °C increased from 2.72 GPa for the pure CER matrix to 2.99–3.24 GPa for the nanocomposites with amino-functionalized POSS nanoparticles. Furthermore, CER/amino-POSS nanocomposites possessed a higher specific surface area, gas permeability (CO_2_, He), and diffusion coefficients (CO_2_) values than those for neat CER, due to an increasing free volume of the nanocomposites studied that is very important for their gas transport properties. Permeability grew by about 2 (He) and 3.5–4 times (CO_2_), respectively, and the diffusion coefficient of CO_2_ increased approximately twice for CER/amino-POSS nanocomposites in comparison with the neat CER network. The efficiency of amino-functionalized POSS in improving the thermal and transport properties of the CER/amino-POSS nanocomposites increased in a raw of reactive POSS containing one primary (APIB-POSS) < eight secondary (*N*PAP-POSS) < one secondary and one primary (AEAPIB-POSS) amino groups. APIB-POSS had the least strongly pronounced effect, since it could form covalent bonds with the CER network only by a reaction of one -NH_2_ group, while AEAPIB-POSS displayed the most highly marked effect, since it could easily be incorporated into the CER network via a reaction of –NH_2_ and –NH– groups with –O–C≡N groups from CER.

## 1. Introduction

Cyanate Ester Resins (CER) constitute a very attractive class of high-performance polymers, which differ from others by a very regular structure of the polymer networks, namely polycyanurates (PCNs), obtained by dicyanate polycyclotrimerization [1,2,3,4,5]. They have received much attention because of their unique combination of physical properties, including a high thermal stability (>400 °C), high glass transition temperature (>270 °C), high fire-radiation and chemical resistance, low water absorption and low outgassing, high adhesion to different substrates, and excellent dielectric properties (ε = 2.64−3.11) [2,3,4]. As a result, CERs are currently used as structural or functional materials in aeronautics, space structures (composite strakes, fins, nose radar domes, heat shields), and printed circuit boards, as well as adhesives [6]. The following companies manufacture CERs for these applications: Cytec Aerospace Materials, Hexcel, Huntsman Advanced Materials, JFC Technologies, Lonza, Henkel, and TenCate Advanced Composites. However, like for most thermosets, their main drawback is brittleness. To overcome this limitation, modifications of CERs have been developed over the past decades, and it is still of great interest. CERs can be modified by many different additives, such as engineering thermoplastics, elastomers, or reactive oligomers [2,3,4,5,7,8,9,10,11,12,13,14] with a formation of semi-IPNs, IPNs and hybrid networks [7,8,9,10,11,12,13,14,15,16,17,18,19,20,21,22,23,24,25,26,27,28,29,30,31,32,33,34]. The improvement of mechanical properties can thus be attained, due to the microphase-separated morphology generation, and especially in the case of a co-continuous morphology.

Unfortunately, the latter improvement is often achieved at the expense of thermal stability. This deficiency is remedied by the synthesis of nanocomposites of CER with montmorillonite (MMT) [35,36,37,38,39,40], carbon nanotubes [41,42], nanostructured aluminum borate [43], ZnO [44], ZrW_2_O_8_ [45], nanosilica [46,47,48], polyhedral oligomeric silsesquioxane (POSS) [49,50,51,52,53,54,55,56,57,58,59,60,61,62,63,64,65,66,67,68,69,70,71,72,73], and other nanofillers. The most pronounced effect on mechanical and thermal properties of CERs is achieved when nanoparticles with organically modified surface are used, as they may react with polymer networks.

POSS represent cage structures with the formula (RSiO_1.5_)_n_ where *n* = 8, 10, 12 and R is hydrogen, reactive, or non-reactive organic groups. Each silicon atom is bonded to three oxygen atoms in a cage and to a single R substituent out of a cage. These substituents improve the compatibility of POSS molecules with polymers or monomers. In the case of reactive R, 3-D POSS molecules with diameters of 1–2 nm, they may graft chemically to polymer structures. New hybrid organic–inorganic CER-based thermosets with hydroxyl- [49,51,52,55,69,72], amino- [50,53], epoxy- [59,60,61,62,63,64,65,66,68,69,70], cyanate- [67], benzoxazinyl- [71] or methacrylate-functionalized [73] POSS units have thus been obtained with improved thermal and mechanical properties.

Recently, thermostable nanocomposites based on densely crosslinked CER doped by 0.01–10 wt.% epoxycyclohexyl-functionalized POSS (ECH-POSS) were synthesized and characterized using TEM, SAXS, EDXS, FTIR, DSC, DMA, TGA, far-IR, and creep rate spectroscopy techniques [60,61]. It was revealed that ultra-low POSS contents (<<1 wt.%) covalently embedded into a CER network substantially changed its nanostructure and properties [60,61,64]. This resulted in changing network dynamics, increasing glass transition temperatures by 20–50 °C, enhancing high temperature elastic and creep resistance properties, and increasing thermal stability under an inert atmosphere at T < 400 °C. The effects decreased, or even became zero or negative, while increasing POSS content, especially from 2 to 10 wt.%, due to arising the structural nanorod- or platelet-like formations and POSS enriched nanodomains. At ultra-low POSS contents, the data obtained suggested basically molecular POSS dispersion, their quasi-periodic spatial distribution in the matrix, and not only chemical modifying the CER network, but also the possible manifestation of the enhanced long-range action of the “constrained dynamics” effect.

The aim of this present work is to synthesize and investigate the structure-properties relationships for nanocomposites of CER filled with amino-functionalized POSS of different reactivities, i.e., possessing different numbers of primary and/or secondary amino-groups. We intend to determine the effect of amino-functionalized POSS nanoparticles on phase structure, morphology, physical properties, and gas permeability of the CER/amino-POSS films. Amino groups are very reactive towards cyanate groups [2,74,75], therefore, during mixing and heating of the amino-POSS nanoparticles with CER monomer, the nanoparticles may react and be chemically incorporated into the growing network to form hybrid organic/inorganic networks with improved physical and chemical properties.

## 2. Materials and Methods

### 2.1. Materials

The CER network was formed using 1,1′-bis(4-cyanatophenyl) ethane (dicyanate ester of bisphenol E, DCBE), under the trade name PRIMASET^®^ LECy, kindly supplied by Lonza (Basel, Switzerland). POSS derivatives, *viz*., aminopropylisobutyl POSS^®^ (APIB-POSS), aminoethyl aminopropylisobutyl POSS^®^ (AEAPIB-POSS), and *N*-phenylaminopropyl POSS^®^ (*N*PAP-POSS) from Hybrid Plastics Inc. (Hattiesburg, MS, USA), were used as received. The chemical structures and basic physical characteristics for these components are given in Table 1.

### 2.2. Synthesis Procedure

The initial DCBE/POSS mixtures were first stirred with a magnetic stirrer (1500 rpm) at *T* ≈ 65 °C over 2 h for POSS dispersion and chemical grafting through the reaction between cyanate groups of CER and amino groups of POSS. Then, the obtained mixtures were poured into a PTFE-coated mold and cured over the temperature range from 25 °C to 300 °C with a heating rate of 0.5 °C/min. All polymer nanocomposites derived from DCBE were synthesized with a constant mass proportion of different amino-POSS equal to 0.1 wt.% with a thickness of 85–115 μm.

### 2.3. Characterization Techniques

Dynamic mechanical thermal analysis (DMTA) was performed using a TA Instruments Q800 analyzer operating with 0.05% of strain amplitude and a frequency of 1 Hz. The samples were heated from −150 °C to 320 °C at a heating rate of 3 °C/min. Loss modulus peaks corresponding to the α relaxation were deconvoluted with the IgorPro 6.38 software. The surface of deconvoluted peaks was calculated via the MultiPeak 1.4 function of this software by fitting the peaks with Gaussian distribution functions. The molar mass between crosslinks *M_c_* was then calculated according to Equation (1) [76]:

(1) $$M_{C}=\frac{E’}{øRTf}$$

where *E*′ is the elastic modulus taken at *T_α_* + 50 °C, *R* is the ideal gas constant equal to 8.314 J/mol·K, *T* = *T_α_* + 50 °C, *f* is the network functionality which for the studied samples was considered equal to 3, and *ϕ* is a factor linked to the network model. In this work, we considered the affine model most suitable for highly crosslinked polymers [77]. For this model *ϕ* = 1.

**Table 1:** Chemical structure and physical characteristics of the components used.

Name	Chemical Structure	Physical Characteristics
Dicyanate ester of bisphenol E, DCBE	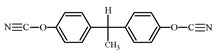	*M* = 264 g·mol^−1^ *T*_m_ = 29 °C *T*_b_ > 240 °C $D_{4}^{20}$ = 1.18 g·cm^−3^ *η* = 75 mPa·s [2]
Aminopropylisobutyl POSS, APIB-POSS	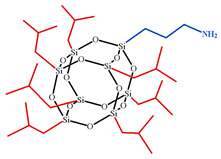	*M*$=875\;g\cdot \mathrm{mol}{-}^{1}\;D_{4}^{20}=1.16\;g\cdot {cm}^{-3}$ $n_{D}^{20}$ = 1.46 [78]
Aminoethyl aminopropylisobutyl POSS, AEAPIB-POSS	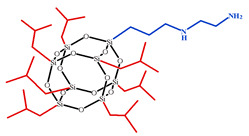	*M* = 918 g·mol^−1^ $D_{4}^{20}=1.17\;g\cdot {cm}^{-3}$ $n_{D}^{20}$ = 1.50 [78]
*N*-Phenylaminopropyl POSS, *N*PAP-POSS	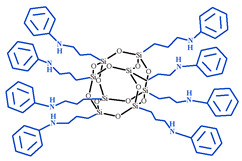	*M* = 1490 g·mol^−1^ $D_{4}^{20}=1.20\;g\cdot {cm}^{-3}$ $n_{D}^{20}$ = 1.57 [78]

The nitrogen sorption measurements were carried out at 77 K (−196 °C) with a Micromeritics GmbH ASAP 2010 analyzer (Unterschleißheim, Germany). The specific surface area (*S*) values were calculated using the Brunauer–Emmett–Teller (BET) method in the relative pressure (P/P_0_) range from 0.05 to 0.3 by Equation (2) [79]:

(2) S=Stw

where *S*_t_ is the total surface area derived from Equation (3) [79], and *w* is the sample mass.

(3) $$S_{t}=\frac{W_{m}\cdot N\cdot A_{cs}}{M}$$

where *W*_m_ is the mass of adsorbate as monolayer, *N* is Avogadro’s number (6.02 × 10^23^ mol^−1^), *A*_cs_ is the adsorbate cross sectional area (16.2 Å^2^ for Nitrogen), and *M* is the molar mass of adsorbate.

The thermal stability of composites was determined by thermogravimetric analysis (TGA) using a Setaram SETSYS evolution 1750 thermobalance, with a platinum pan under 20 mL/min argon flow at a heating rate of 20 °C/min from 50 °C to 650 °C. The initial mass of the samples was equal to about 10 mg in all the cases.

Differential scanning calorimetry (DSC) with the Perkin-Elmer Diamond DSC apparatus was used for estimating glass transition temperatures, *T*_g_, at the half-height of a heat capacity step and glass transition onset temperatures, *T*_g onset_, in the composites. The second scans were performed with the heating rate of 40 °C/min over the temperature range from 20 to 350 °C in a nitrogen atmosphere. The temperature scale was calibrated with pure Indium (*T*_m_ = 156.6 °C).

Permeation measurements were performed at 20 °C for helium (He) and carbon dioxide (CO_2_) with respective kinetic diameters of 2.6 and 3.3 Ǻ [80]. The CER-based samples, with a useful area of 3 cm^2^ and a constant thickness around 150 μm, were placed between the upstream and downstream compartments of the permeation cell. A secondary vacuum desorption step was performed prior to each permeation experiment. The permeation measurements were carried out under an upstream pressure, *P*_1_, equal to 3 bars. The downstream pressure, *P*_2_, was measured as a function of time. The permeability coefficient, *P*, was calculated from the slope of the linear time dependence of *P*_2_ in a steady state, and the diffusion coefficient, *D*, was deduced from the time lag, *θ*, as determined by the extrapolation of the steady-state line on the time axis (Equation (4) [80]):

(4) $$D=\frac{L^{2}}{6\theta }$$

where *L* is the film thickness. *D* was expressed in cm^2^·s^−1^ and *P* in barrer (with 1 barrer = 10^−10^ ${cm}^{3}(STP)\cdot cm/({cm}^{2}\cdot s\cdot cm\mathrm{Hg}$)).

Water sorption isotherms of the different films were determined at 25 °C by using the dynamic vapor sorption analyzer, DVS Advantage (London, United Kingdom). Each sample was pre-dried in the DVS Advantage by exposure to dry nitrogen until the equilibrated dry mass was obtained (*m*_0_). A partial pressure of vapor (*Pi*) was then established within the apparatus by mixing controlled amounts of dry and saturated nitrogen and the mass of the sample (*m*_t_) was followed as a function of time. The mass of the sample at equilibrium (*m*_eq_) was considered to be reached when changes in mass with time (*d*m/*d*t) were lower than 2 × 10^−4^ mg·min^−1^ for at least 5 min. Then, vapor pressure was increased in suitable activity (*a_w_*: 0, 0.2, 0.5, 0.7, and 0.9, respectively). The value of the mass gain at equilibrium (*G*) was defined by Equation (5) [80]:

(5) $$G=\frac{m_{eq}-m_{0}}{m_{0}}$$

For each water activity (*a*_w_), it permitted to plot the water sorption isotherm. The precision on the values of *G* was estimated to be better than 5%.

The diffusion coefficient (*D*) was determined according to Equation (6) [80]:

(6) D=0.04909·L2t12

where *t*_1/2_ is the half sorption time and *L* is the sample thickness.

## 3. Results and Discussions

It is well known that cyanate groups of CERs can readily react with hydrogen-containing functional groups, such as –OH, –NH_2_, and –NH–, the reaction with –NH_2_ and –NH– groups occurring at temperature of ~30 °C and ~65°C, respectively [74,75]. Recently, these reactions have been applied for the chemical incorporation of different functionalized nanofillers into in situ growing CER networks to create high performance thermostable CER-based nanocomposites [40,46,47,48,50,51]. Cho et al. [50] confirmed chemical incorporation of amino-POSS into the CER (PT-30) network through the reaction of the –O–C≡N groups of CER with –NH_2_ groups of amino-POSS with the formation of RNHC(=NH)OR fragments that were evidenced by FTIR with a stretching band at 1640 cm^−1^. In a previous study [40], some of us observed the same band as well in the cured CER/amino-MMT nanocomposites.

**Figure 1:**
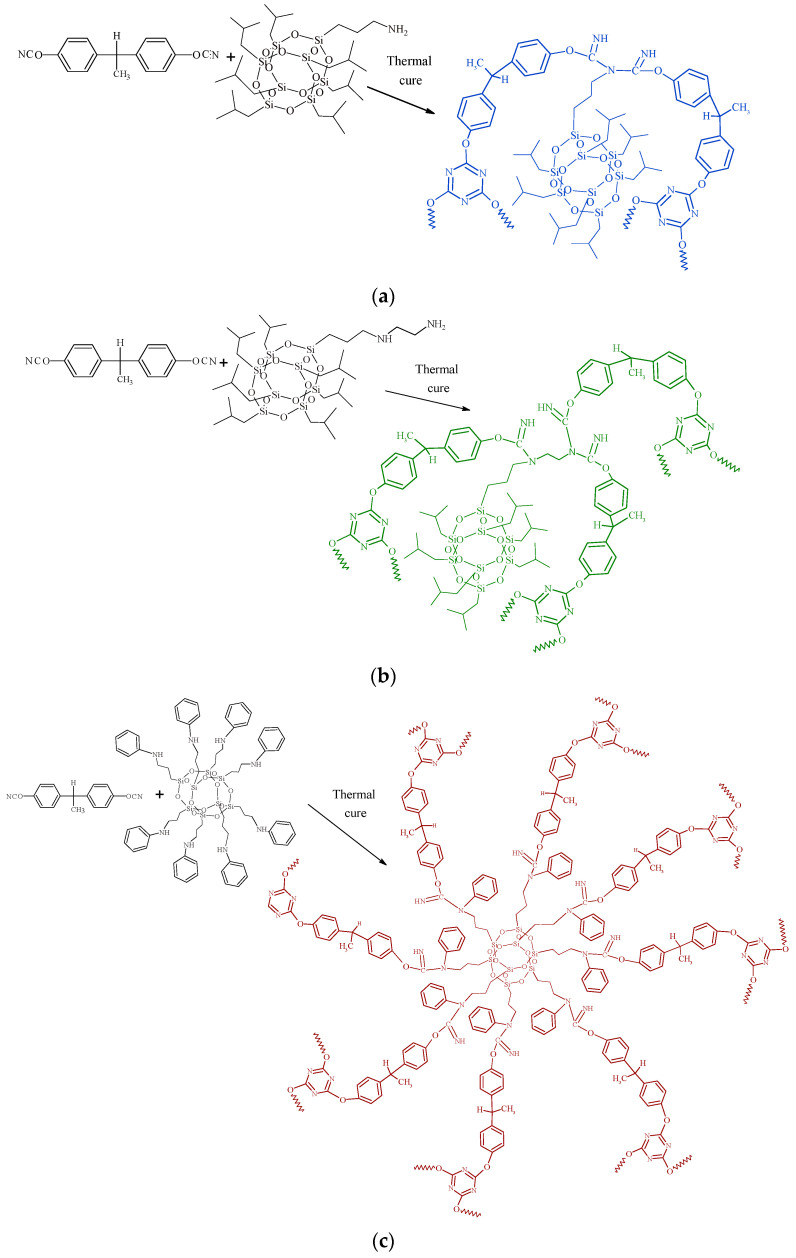
Schematic presentation of chemical embedding to growing CER network of different amino-POSS nanoparticles: (**a**) APIB-POSS, (**b**) AEAPIB-POSS, and (**c**) *N*PAP-POSS.

In this present work, the initial stage of amino-POSS chemical grafting or incorporation into the CER network is schematically shown in Figure 1. Due to differences in the functionality of POSS nanoparticles, it is possible to envision a large variety in the design of the synthesized CER/amino-POSS nanocomposites.

### 3.1. Investigation of Viscoelastic Properties by DMTA

**Figure 2:**
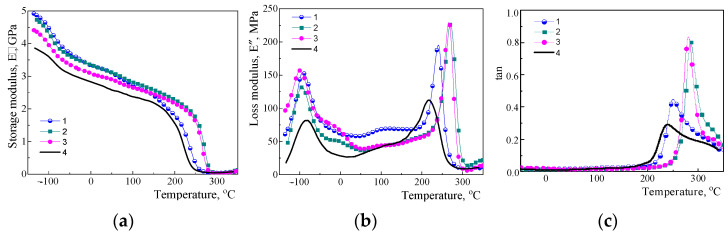
Temperature dependence (at 1 Hz) of (**a**) storage modulus, *E*′, (**b**) loss modulus, *E*″, and (**c***)* tan δ for (1) CER/APIB-POSS, (2) CER/AEAPIB-POSS, (3) CER/*N*PAP-POSS nanocomposites, and (4) neat CER network.

The influence of embedding amino-POSS nanoparticles into polycyanurate networks on the viscoelastic properties of the nanocomposites synthesized was investigated by using DMTA. Figure 2 shows the temperature dependence of storage modulus, *E*′ (Figure 2a), loss modulus, *E*″ (Figure 2b), and tan δ (Figure 2c) for the neat CER network and for the nanocomposites with different amino-POSS. Table 2 displays the corresponding viscoelastic characteristics.

One could see a significant influence of the addition of different types of reactive amino-POSS nanoparticles on viscoelastic properties for all the CER/amino-POSS nanocomposites synthesized. Indeed, the values of storage modulus *E*′, the intensities of loss modulus maxima *E*″, and the values of α transition temperatures *T*_α_ for the CER matrix substantially changed upon introduction of the nanoparticles (see Table 2). All the above-mentioned changes evidenced the essential differences in hybrid CER/amino-POSS networks depending on the structure of the amino-POSS used, namely the number and the reactivity of amino groups on the POSS cage surface. Amino-POSS molecules could graft from one side (in the case of APIB-POSS having one amino group) or incorporate inside (in the case of AEAPIB-POSS or *N*PAP-POSS having two or eight amino groups, respectively) the CER, thus, resulting in the formation of hybrid organic-inorganic CER/POSS nanocomposites. Therefore, during the synthesis of CER/amino-POSS nanocomposites, the formation of mixed microphases with different contents of chemically grafted or incorporated amino-POSS nanoparticles in the CER matrix (i.e., microphases with different mobility of kinetic segments of macromolecules) took place. Figure 2 shows that loading nanofiller into the CER network led to a significant increase of storage modulus, *E*′, values in the temperature region was investigated. This fact evidenced the strengthening of the elastic properties associated with the polymer matrix, probably due to the well distributed relatively large nanoparticles of rigid POSS along the segments of the polymer chains as additional inorganic junctions, which hindered and restricted the movement of these segments. The uniform distribution of amino-POSS nanoparticles in the CER network without their aggregation was undoubtedly achieved due to two main reasons: (i) a sufficiently low (0.1 wt.%) nanofiller content, and (ii) the use of a special preheating procedure (before the main synthesis) for ensuring chemical interactions between the functional groups of CER and amino-POSS. Each POSS nanoparticle was thus surrounded by CER molecules transforming into the polymer network at further high temperature curing, without agglomeration. As a result, the high crosslink density organic-inorganic hybrid polymer network was formed obviously without significant defects.

Recently, Zhang et al. [81] mathematically processed the curves *E*″ = *f* (*T*) for an individual CER using inverse convolution by the contributions of single relaxation processes. Four distinct peaks corresponding to the main α-relaxation of a higher intensity at a higher temperature, and secondary γ- and β-relaxations of lower intensities at lower temperatures were detected. From Figure 2b, one could see that both neat CER and all CER/amino-POSS nanocomposites exhibited broad γ-relaxation assigned to the phenylene and methyl groups rotations present in the links between the planar six-member three-arm cyanurate rings of CER structure [82] with a maximum at *T* ≈ −85 °C and in the temperature range from −90 °C to −100 °C, correspondingly. Secondary relaxations appeared with a maximum at *T*_β_ (from −43 to −11 °C, Table 2) and at *T*_β′_ ≈ 100 °C, corresponding to the β and β′ relaxations associated with the mobility of the chain fragments between the crosslink sites of the CER network [83]. One could see from Figure 2 and Table 2 that the CER/amino-POSS nanocomposites possessed higher values of *T*_α_ compared to the unfilled CER network. Herewith, loading the APIB-POSS into the CER network resulted in a temperature shift towards higher *T*_α_ values by 18 °C, whereas the addition of *N*PAP-POSS and AEAPIB-POSS sharply increased the *T*_α_ values by 44 °C and 50 °C, respectively (see Table 2). Thus, the introduction of amino-POSS nanoparticles chemically grafted/incorporated into the CER networks led to the formation of new hybrid crosslinking sites. As a result, the additional steric obstacles reduced the amplitude of the spatial mobility of the kinetic segments of the macromolecules of the CER network, which caused an increase in the value of *T*_α_, thus confirming conclusions of Bershtein et al. [61] about the enhanced long-range action of the “constrained dynamics” effect. Therefore, in the presence of amino-POSS, the values of *T*_γ_ decreased, whereas the values of *T*_β_, *T*_α_, *E*′, and *M*_c_ increased, evidencing that amino-POSS behaved as both thermal and mechanical antiplasticizers i.e., reinforcements.

The curves for *E*″ and tan δ seemed to be in contradiction to what the *E*′ and *T_α_* values showed. Indeed, an apparent increase in the *E*″ and tan δ peak intensities is observed for α relaxation in the presence of APIB-POSS, *N*PAP-POSS, and AEAPIB-POSS, whereas a “classical” anti-plasticization effect (i.e., increase of *T_α_*) would tend toward the reduction of such peak intensities, since the material would be considered stiffer. However, it should be considered that not only the intensity of *E*″ and tan δ peaks represent the viscoelastic behavior of a polymer, but also the peak amplitude and half-width. This is due to the fact that the main α relaxation is a heterogeneous phenomenon spanning a range of temperatures. In order to really distinguish the influence of added nanofillers on the viscoelastic behavior of CER networks, the surfaces of the *E*″ peaks, representing the total modulus loss during the whole α relaxation phenomenon should be compared. The *E*″ peaks observed for the α relaxation were deconvoluted and integrated as detailed in the Experimental section. A single Gaussian function was considered for CER/APIB-POSS, CER/*N*PAP-POSS, and CER/AEAPIB-POSS samples whereas two Gaussian functions were considered for neat CER. The computed surfaces are summarized in Table 2.

Table 2 shows that CER/APIB-POSS and neat CER had similar *E*″ peak surfaces, that of CER/APIB-POSS being slightly lower. The reduction of the *E*″ peak surfaces was further observed for CER/*N*PAP-POSS, and CER/AEAPIB-POSS nanocomposites. This meant that the introduction of APIB-POSS, *N*PAP-POSS, and AEAPIB-POSS actually reduced the viscous behavior of CER, as the surface of these peaks correspond to the loss due to the viscoelastic nature of polymers during the α relaxation phenomenon, as detailed in the previous paragraph. As such, the observed increase in *E*′′ and tan δ intensities was not due to an increase in a viscous behavior but could be correlated to a more homogeneous α relaxation phenomenon induced by the presence of the amino-based nanofiller. Finally, it could be concluded that CER/APIB-POSS, CER/*N*PAP-POSS, and CER/AEAPIB-POSS nanocomposites induced a more homogenous α relaxation phenomenon with higher *T_α_* values, and an enhanced nanocomposite elastic behavior compared to unfilled CER network.

### 3.2. Investigation of Thermophysical Properties by DSC

**Figure 3:**
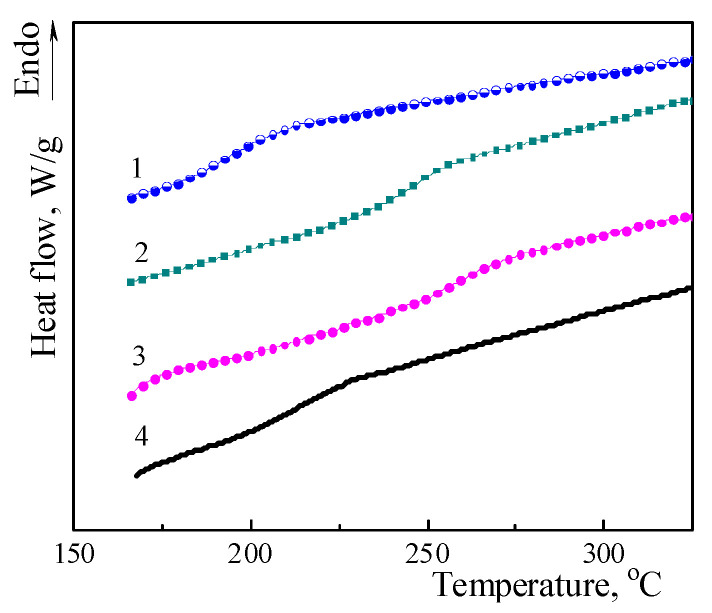
DSC thermograms (2nd heating scan) for (1) CER/APIB-POSS, (2) CER/AEAPIB-POSS, (3) CER/*N*PAP-POSS nanocomposites, and (4) neat CER network.

The effect of chemically embedded amino-POSS nanoparticles into the CER network on the thermophysical properties of CER/amino-POSS nanocomposites was also studied by DSC. Figure 3 shows corresponding DSC thermograms, and Table 3 displays the obtained thermophysical characteristics (*T*_g onset_, *T*_g_, and Δ*C*_p_). It is noteworthy that DSC data were in good agreement with DMTA results. For all the CER/amino-POSS nanocomposites, only a single *T*_g_ value was evidenced that meant that all the samples studied had an amorphous structure. However, the *T*_g onset_, *T*_g_, and Δ*C*_p_ values of nanocomposites varied significantly depending on the functionality of amino-POSS embedded into the CER matrix. Meanwhile, introducing APIB-POSS nanoparticles led to the negligible growth of *T*_g_ (by 2 °C) of polycyanurate matrix. However, adding *N*PAP-POSS and AEAPIB-POSS shifted *T*_g_ values to higher temperatures by 45 °C and 55 °C, respectively (cf. Table 3). Zhang et al. [81,82] attributed the increase in *T*_g_ of nanocomposites as compared to the unfilled (neat) polymer with the suppression of the polymer chain mobility by POSS molecules (cages) and Bershtein et al. [61] explained this fact by the possible manifestation of the enhanced long-range action of the “constrained dynamics” effect.

### 3.3. Investigation of Thermal Stability by TGA

**Figure 4:**
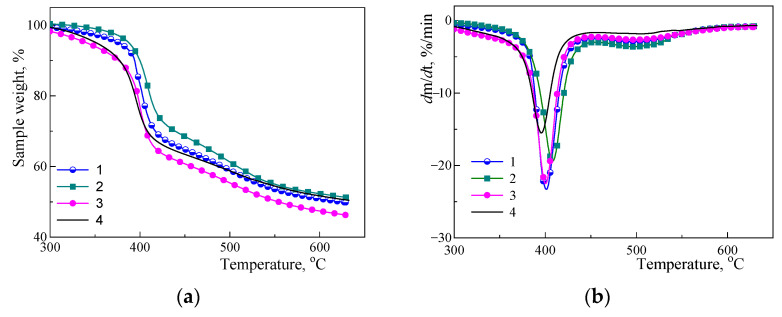
Typical (**a**) TGA and (**b**) DTG curves (in argon) for (1) CER/APIB-POSS, (2) CER/AEAPIB-POSS, (3) CER/*N*PAP-POSS nanocomposites, and (4) neat CER network.

The effect of the different amino-POSS used on thermal stability of the nanocomposites synthesized was studied by TGA. Figure 4 shows TGA (Figure 4a, in argon) and corresponding DTG (Figure 4b) curves for the CER/amino-POSS nanocomposites (Figure 4, curves 1–3) compared to that for the neat CER network (Figure 4, curve 4). The corresponding thermal characteristics are summarized in Table 4. A strong influence of even such a low content (0.1 wt.%) of the selected nanofillers on thermal stability of CER/amino-POSS nanocomposites formed in situ was clearly observed. Indeed, the improved thermal stability was shown when the APIB-POSS or AEAPIB-POSS were used in a contrast to *N*PAP-POSS.

Two simultaneous processes could occur during CER/amino-POSS nanocomposite formation: (i) the densely crosslinked polymer network with additional hybrid inclusions/junctions was formed, due to chemical grafting/incorporation of thermostable POSS nanoparticles into the CER network that led to an improvement of thermal characteristics of CER-based nanocomposites, and (ii) some defects were generated in the CER network, due to the existence of POSS nanoparticles with diameters of 1–2 nm inside the growing CER matrix that could hinder the formation of the regular CER network and weaken its thermal properties.

One could see that neat CER and CER/amino-POSS nanocomposites were characterized by two stages of decomposition. The main stage was in a region of ~374–440 °C related to the degradation of cyanurate skeleton [2,3], and a second stage at 467–531 °C. The residual char was determined to be ~46–51% at 630 °C. Nevertheless, the thermal stability of the nanocomposites increased when only 0.1 wt.% of different amino-POSS were loaded compared to the unfilled CER matrix. Thus, one could conclude that the temperature of the intensive decomposition onset for the unfilled CER matrix was quite high (*T*_d1 onset_ = 374 °C), and it increased by ≈9–15 °C when introducing the amino-POSS nanoparticles. The temperature of the maximal degradation rate *T*_d1 max_ shifted to higher temperatures by ≈5–12 °C as well depending on the functionality of the amino-POSS used. It should be pointed out that when loading both APIB-POSS with one primary amino group and *N*PAP-POSS with eight secondary amino groups into the CER matrix, all the thermal characteristics increased by 5–10 °C compared to the neat CER network. More interestingly, the greatest impact on thermal stability (increasing by 12–15 °C) was observed with loading of AEAPIB-POSS with one secondary and one primary amino group. It was obvious that primary amino groups had a reactivity higher than that of secondary amino groups, which resulted in a higher degree of POSS nanoparticles grafting to CER network. In conclusion, the highest effect was logically observed for the AEAPIB-POSS, where the effect of the primary amino group was further enhanced by the presence of a secondary amino group.

The increase in thermal stability of the nanocomposites studied was undoubtedly associated with the creation of numerous additional organic-inorganic nodes in the hybrid CER/amino-POSS network and, probably with the formation of an additional network of hydrogen bonds between the functional groups of POSS and CER. It is noteworthy that the ultra-low amount (0.1 wt.%) of amino-POSS and the special preheating/mixing synthesis procedure used for sample preparation prevented the aggregation of POSS nanoparticles and promoted efficient dispersion and chemical incorporation of the nanoparticles into the CER matrix. The higher the number and reactivity (with respect to the CER cyanate groups) of the amino groups in amino-POSS, the stronger the effect of POSS on the thermal stability of the nanocomposite. It is clear that additional energy was required to destroy multiple hybrid cross-links in the nanocomposites studied. Obviously enough, at high POSS contents, due to the increased aggregation of nanoparticles and occurrence of defects in the highly regular structure of the CER network, a decrease in the thermal stability of the samples could be observed [58,61,64].

It was of interest to compare the properties of CER/amino-POSS nanocomposites studied in this work with experimental data published elsewhere [50,53,82]; the corresponding comparative data are given in Table 5. Unfortunately, due to significant differences in the composition of the samples under investigation (CER matrix, component content), synthesis methods, as well as experimental conditions of analysis, it was impossible to directly compare the properties of the nanocomposites from the different references. However, some general conclusions could be drawn below.

First, it was clear that the CER/amino-POSS nanocomposites studied in this work were synthesized and studied for the first time. Second, the concentration of the nanofillers in the nanocomposites studied was 10–200 times lower than in previous reports [50,53,82], where the concentration of amino-POSS varied from 1 to 20 wt.%. Third, the strong synergistic effect of ultra-small amounts (0.1 wt.%) of amino-POSS on the viscoelastic properties and thermal stability of the synthesized nanocomposites was also demonstrated for the first time. For example, the greatest increase in the *T*_g_ value by 52 °C (compared to the unfilled CER) was recorded in this work for CER/AEAPIB-POSS nanocomposite (Table 5), while for the nanocomposites described in the literature, the maximum increase in the *T*_g_ value was equal to 31 °C only (PT-15/OAP-POSS = 99/1 wt.%, [53]) and the maximum increase in *T*_g(end)_ value was equal to 43 °C only (PT-15/OAPr-POSS = 80/20 wt.%, [82]). Additionally, for the nanocomposites herein investigated, the values of moduli *E*′ at 40 °C were 1.5–2.3 times higher than those for the nanocomposites previously studied (Table 5) [52,53,82]. We also found a significant effect of low concentrations of amino-POSS on the thermal stability of the nanocomposites studied: for CER/APIB-POSS and CER/AEAPIB-POSS, the *T*_d5%_ value increased by 25 °C and 36 °C (in comparison to neat CER network), respectively. Thus, one could conclude that using ultra-low amounts of amino-POSS additives was an effective way to improve the properties of novel nanocomposites based on CER matrixes of different chemical structures.

### 3.4. Investigation of Gas Transport Properties

**Figure 5:**
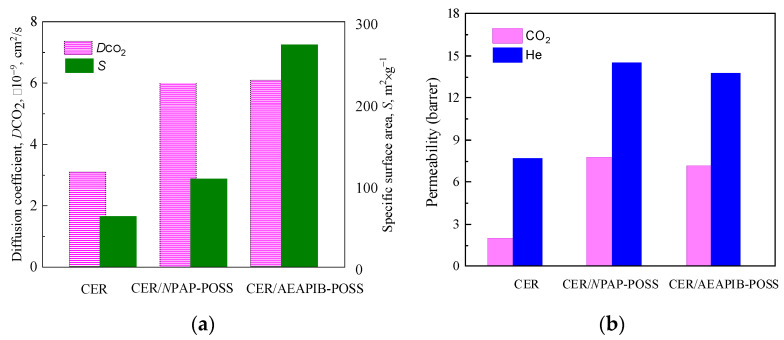
Specific surface area (**a**), diffusion coefficient (**a**), and permeability coefficient (**b**) for neat CER and CER/amino-POSS films under investigation.

The specific surface area (*S*) values for all the synthesized samples were determined from nitrogen adsorption/desorption isotherms by using the BET method. The *S* value for the neat CER network was equal to around 62 m^2^/g, whereas the introduction of amino-POSS nanoparticles increased the values of specific surface area to 105, 108, and 272 m^2^/g for CER/APIB-POSS, CER/*N*PAP-POSS, and CER/AEAPIB-POSS nanocomposites, respectively (Figure 5a).

Gas transport properties for neat CER and CER/amino-POSS nanocomposites were determined, and the results obtained are depicted in Figure 5b. He and CO_2_ permeability increased, respectively, between ≈2 and 3.5–4 times, and the diffusion coefficient of CO_2_ increased approximately twice for CER/amino-POSS nanocomposites in comparison with the neat CER network. These results were in a good correlation with the above reported data on the specific surface area. Obviously, the introduction of amino-POSS nanoparticles led to an increasing free volume of the nanocomposites studied that represented additional diffusion paths for gas transport [84,85]. Formation of a looser structure in hybrid CER-based nanocomposite networks was also confirmed by increasing Δ*C*_p_ values (see DSC data in Table 3) and increasing *M*_c_ values (see DMTA data in Table 2) with incorporation of amino-POSS nanoparticles into the CER network.

**Figure 6:**
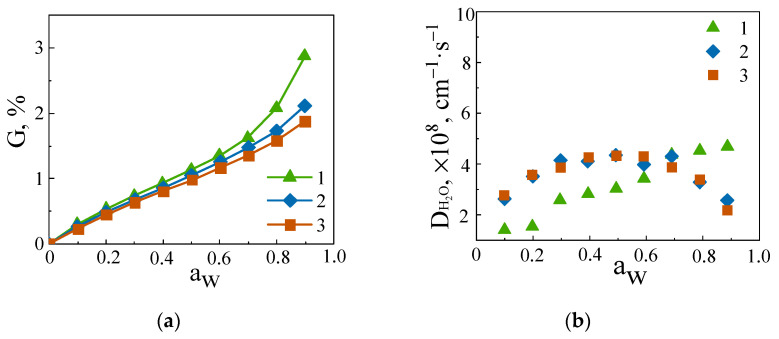
Water sorption (*G*) isotherms (**a**) and evolution of the water diffusion (*D*) (**b**) for neat CER (1), CER/*N*PAP-POSS (2), and CER/AEAPIB-POSS (3) nanocomposites as a function of water activity.

Figure 6a displays the evolution of the water uptake (*G*) for the neat CER network and CER/*N*PAP-POSS and CER/AEAPIB-POSS nanocomposites. The water uptakes remained rather low (less than 3 wt.%) as usually observed in the case of thermosets [84,86]. The water sorption isotherms were of the BET II type. The slight curvature observed at low water activity (below a_w_ = 0.2) was related to the presence of water molecules strongly linked to the polar groups contained in the materials, and the positive deviation from linearity evidenced at high water activity indicated a water clustering phenomenon. One could observe a small decrease in the water uptake at high water activity for CER/amino-POSS nanocomposite samples compared to neat CER, meaning that the presence of POSS hindered water clustering (Figure 6a). The evolution of the water diffusion coefficient (*D*) values of the samples studied as a function of the water activity is presented in Figure 6b. The *D* values measured at low water activity were higher for the CER/amino-POSS films with respect to neat CER. This trend was the same as that observed for gas transport, and it was related to the higher free volume contained in the CER/amino-POSS films. It could also be observed that the profile of water diffusion of neat CER network was different from that of CER/*N*PAP-POSS and CER/AEAPIB-POSS. It seemed that the higher clustering phenomenon observed at high water activity for neat CER induced a slight plasticization effect with an increase in *D* values, whereas the more limited clustering effect taking place in CER/amino-POSS films led to a small decrease in *D* values, as usually observed in networks [86]. However, *D* variations remained small in both cases.

## 4. Conclusions

Nanocomposites of cyanate ester resin (CER) filled with different types of reactive amino functionalized POSS were synthesized and characterized. For the first time, a comparative analysis of the effect of ultra-low concentrations of amino-functionalized POSS with different numbers of primary and/or secondary amino groups covalently bonded to CER-based polymer networks on basic properties of resulting nanocomposites was completed. Improvement of thermal, mechanical, and gas transport properties was evidenced. It was found that the addition of a small quantity (0.1 wt.%) of amino-POSS chemically grafted to the CER network led to a significant increase in the thermal stability of the CER matrix. Namely, the degradation temperature onset, *T*_d onset_, and the temperature of maximal degradation rate, *T*_d max_*,* shifted to higher temperatures by ≈12–15 °C, depending on the type of amino-POSS. The significant growth of glass transition and α relaxation temperatures, *T*_g_ and *T*_α_, by 45–55 °C of the CER matrix with the loading of nanofillers was demonstrated by means of DSC and DMTA, respectively. The essential increase in storage modulus values in the temperature region investigated was observed as well. Formation of looser structures in hybrid CER-based nanocomposite networks was also confirmed by increasing Δ*C*_p_ (DSC data) and increasing *M*_c_ values (DMTA data) with incorporation of amino-POSS nanoparticles into the CER network.

It was shown that CER/amino-POSS nanocomposites possessed specific surface area, gas permeability (CO_2_, He), and diffusion coefficients (CO_2_ and H_2_O at low activity) higher than that for neat CER. The efficiency of amino-functionalized POSS in improving the thermal and gas transport properties of the CER/amino-POSS nanocomposites increased in a raw of POSS containing one primary amino group (APIB-POSS) < eight secondary amino groups (*N*PAP-POSS) < one secondary and one primary amino groups (AEAPIB-POSS). APIB-POSS had the least strong effect, since it could form covalent bonds with CER network only by reaction of one –NH_2_ group. *N*PAP-POSS could connect to the CER network through reaction of 8 –NH– groups but reactivity of –NH– groups was quite low, so its effect was slightly diminished. AEAPIB-POSS had the strongest effect, since it could easily be incorporated into the CER network via reaction of one –NH_2_ and one –NH– groups with –O–C≡N groups of CER.

## Figures and Tables

**Table 2:** Viscoelastic properties (DMTA data) for CER/amino-POSS nanocomposites and neat CER network.

Sample	*T*_γ_, °C	*T*_β_, °C	*T*_α_, °C	*E*′ at 25 °C, GPa	*M*_c_, g/mol	Surface of *E*″ Peak at *T*_α_, MPa/K	Height of tan *δ* (at *T*_α_)
(1) CER/APIB-POSS	−92	−43	241	3.24	49	13.61	0.45
(2) CER/AEAPIB-POSS	−95	−11	270	3.23	83	9.83	0.81
(3) CER/*N*PAP-POSS	−99	−28	266	2.99	48	12.92	0.84
(4) Neat CER network	−85	−37	218	2.72	31	13.83	0.29

**Table 3:** DSC data for CER/amino-POSS nanocomposites and neat CER network.

Sample	*T*_g onset_, °C	*T*_g_, °C	Δ*C*_p_, J·g^−1^·K^−1^
(1) CER/APIB-POSS	204	216	0.363
(2) CER/AEAPIB-POSS	257	269	0.415
(3) CER/*N*PAP-POSS	249	259	0.314
(4) Neat CER network	203	214	0.351

**Table 4:** Thermal stability of CER/amino-POSS nanocomposites and neat CER network.

Sample	*T*_d5%_^(a)^, °C	*T*_d max_^(b)^, °C	Δ*m ^(^*^c)^, %	*m*_ash_ ^(d)^, %
(1) CER/APIB-POSS	377	401	23	50
(2) CER/AEAPIB-POSS	388	408	18	51
(3) CER/*N*PAP-POSS	341	401	23	46
(4) Neat CER network	352	396	21	51

^(a)^ *T*_d5%_ is the temperature of 5% mass loss; ^(b)^ *T*_d max_ is the temperature value of maximal degradation rate; ^(c)^ Δ*m* is the mass loss at maximal degradation rate; ^(d)^ *m*_ash_ is the ash content at *T* = 630 °C.

**Table 5:** Comparative table for some properties of CER/amino-POSS nanocomposites.

CER Matrix	Amino-POSS	Curing Schedule	POSS Content, wt.%	*T*_g_, °C	*E*′, GPa (at 40 °C)	*T*_g_’s Method Condition	*T*_d5%_, °C, TGA, 20 °C/min	Ref.
Bisphenol-F based CER (PT-15)	DDAP-POSS	188 °C/120 min	1	~225	~2.69	DMTA, 10 Hz (tan δ data)	-	[50]
Bisphenol-F based CER (PT-15)	-	188 °C/120 min + to 250 °C at 5 °C/min + 250 °C/180 min	0	305	1.50	DMTA, 1 Hz (tan δ data)	-	[53]
	OAP-POSS		1	336	1.61		-	
	OAP-POSS		3	300	2.12		-	
	OAP-POSS		5	258	1.41		-	
Bisphenol-F based CER (PT-15)	CPPHCP-POSS	188 °C/120 min + to 250 °C at 5 °C/min + 250 °C/180 min + 300 °C/30 min	1	323	1.96	DMTA, 1 Hz (tan δ data)	-	[53]
	CPPHCP-POSS		3	320	2.01		-	
	CPPHCP-POSS		5	331	1.85		-	
	CPPHCP-POSS		10	333	1.66		-	
Bisphenol-A based CER (BADCy)	-	120 °C/60 min + 150 °C/60 min + 180 °C/120 min + 200 °C/240 min	0	268	-	DSC, 10 °C/min (*T*_g(end)_)	*-*	[82]
	OAPr-POSS		1	285	-		-	
	OAPr-POSS		5	306	-		-	
	OAPr-POSS		10	308	-		-	
	OAPr-POSS		20	311	-		-	
Bisphenol-E based CER (LECy)	-	65 °C/120 min (1500 rpm) + 20 °C to 300 °C at 0.5 °C/min	0	218	2.60	DMTA, 1 Hz, 3 °C/min (tan δ data)	352	this manuscrip
	APIB-POSS		0.1	241	3.17		377	
	AEAPIB-POSS		0.1	270	3.17		388	
	NPAP-POSS		0.1	266	2.94		341	

## Data Availability

Data are contained within the article.

## Nomenclature

*A*_cs_	adsorbate cross sectional area
*a*_w_	water activity
AEAPIB-POSS	aminoethyl aminopropylisobutyl polyhedral oligomeric silsesquioxane
amino-POSS	amino-functionalized polyhedral oligomeric silsesquioxane
APIB-POSS	aminopropylisobutyl polyhedral oligomeric silsesquioxane
BET	Brunauer–Emmett–Teller
CER	Cyanate Ester Resin
CPPHCP-POSS	3-cyanopropylheptacyclopentyl polyhedral oligomeric silsesquioxane
*D*	diffusion coefficient
DCBE	dicyanate ester of bisphenol E
DDAP-POSS	dodecaaminophenyl polyhedral oligomeric silsesquioxane
$D_{4}^{20}$	density at *T* = 20 °C
DMTA	dynamic mechanical thermal analysis
DSC	differential scanning calorimetry
DTG	derivative thermogravimetric analysis
DVS	dynamic vapor sorption analyzer
ECH-POSS	epoxycyclohexyl-functionalized polyhedral oligomeric silsesquioxane
*E*′	storage modulus
*E*″	loss modulus
EDXS	energy dispersive X-ray spectrometry
*f*	network functionality
far-IR	far infrared spectroscopy
FTIR	Fourier transform infrared spectroscopy
*G*	water sorption
IPN	interpenetrating polymer network
*L*	film thickness
*M*	molar mass
*m*_ash_	ash content at *T* = 630 °C
*M*_c_	molar mass between crosslinks
*m*_eq_	mass of the sample at equilibrium
*m*_t_	mass of the sample
*m*_0_	equilibrated dry mass
MMT	montmorillonite
*N*	Avogadro’s number
*N*PAP-POSS	*N*-phenylaminopropyl polyhedral oligomeric silsesquioxane
OAP-POSS	octaaminophenyl polyhedral oligomeric silsesquioxane
OAPr-POSS	octaaminopropyl polyhedral oligomeric silsesquioxane
*P*	permeability coefficient
*P_i_*	partial pressure of vapor
*P*_1_	upstream pressure
*P*_2_	downstream pressure
PCN	polycyanurate
POSS	polyhedral oligomeric silsesquioxane
PT-15	Bisphenol-F Cyanate Ester Resin
PTFE-coated	polytetrafluoroethylene coated
*R*	ideal gas constant
*S*	specific surface area
*S*_t_	total surface area
SAXS	small-angle X-ray scattering
*T*_b_	boiling temperature
*T*_d max_	temperature value of maximal degradation rate
*T*_d5%_	temperature of 5% mass loss
*T*_g_	glass transition temperature
*T*_g onset_	glass transition onset temperature
*T*_g(end)_	glass transition end temperature
*T*_m_	melting temperature
*T*_α_	temperature of α relaxation
*T*_β_	temperature of β relaxation
*T*_γ_	temperature of γ relaxation
*t*_1/2_	half sorption time
TEM	transmission electron microscopy
TGA	thermogravimetric analysis
*w*	sample mass
*W*_m_	mass of adsorbate as monolayer,
Δ*C*_p_	heat capacity
Δ*m*	mass loss at maximal degradation rate
ε	dielectric permittivity
*η*	dynamic viscosity
$n_{D}^{20}$	refractive index
*θ*	time lag
*ϕ*	factor linked to the network model
